# Simultaneous and Spatially-Resolved Analysis of T-Lymphocytes, Macrophages and PD-L1 Immune Checkpoint in Rare Cancers

**DOI:** 10.3390/cancers14112815

**Published:** 2022-06-06

**Authors:** Karina Cereceda, Nicolas Bravo, Roddy Jorquera, Roxana González-Stegmaier, Franz Villarroel-Espíndola

**Affiliations:** 1 Translational Medicine Laboratory, Department of Cancer Research, Instituto Oncologico Fundacion Arturo Lopez Perez, Santiago 8320000, Chile; karina.cereceda@falp.org (K.C.); roddy.jorquera@falp.org (R.J.); roxana.gonzalez@falp.org (R.G.-S.); 2 Medical Informatics Unit, Department of Cancer Research, Instituto Oncologico Fundacion Arturo Lopez Perez, Santiago 8320000, Chile; nicolas.bravo@falp.org

**Keywords:** tumor microenvironment, PD-L1, TILs, immunofluorescence, cell segmentation, rare cancer

## Abstract

**Simple Summary:**

To study various biomarkers, it is necessary to analyze multiple tissue sections through serial histological sections, which is challenging when only a small tissue sample is available. In this work we have developed a validated and objective method for combined biomarker immunostaining and its digital image analysis using open informatics tools, which is necessary for comprehensive understanding of the tumor microenvironment in rare cancers and in cases of limited samples with very significant clinical features.

**Abstract:**

Penile, vulvar and anal neoplasms show an incidence lower than 0.5% of the population per year and therefore can be considered as rare cancers but with a dramatic impact on quality of life and survival. This work describes the experience of a Chilean cancer center using multiplexed immunofluorescence to study a case series of four penile cancers, two anal cancers and one vulvar cancer and simultaneous detection of CD8, CD68, PD-L1, Cytokeratin and Ki-67 in FFPE samples. Fluorescent image analyses were performed using open sources for automated tissue segmentation and cell phenotyping. Our results showed an objective and reliable counting of objects with a single or combined labeling or within a specific tissue compartment. The variability was below 10%, and the correlation between analytical events was 0.92–0.97. Critical cell phenotypes, such as TILs, PD-L1+ or proliferative tumor cells were detected in a supervised and unsupervised manner with a limit of detection of less than 1% of relative abundance. Finally, the observed diversity and abundance of the different cell phenotypes within the tumor microenvironment for the three studied tumor types confirmed that our methodology is useful and robust to be applicable for many other solid tumors.

## 1. Introduction

Anal, vulvar, and penile cancer are tumors of external genitalia considered as rare due to their low incidence among the global population, showing an incidence of 0.65, 1.2 and 0.92 cases per 100,000 habitants in 2020, respectively [1,2]. In Chile, these types of cancer are also rare, with between 100 and 140 new cases diagnosed per year. During 2020, the cases of anal, vulvar and penile cancer were 134, 128 and 93, respectively, with a prevalence at five years of 1.8, 3.5, and 2.6 per 100,000 Chileans [1].

All of these tumors have in common their development from a premalignant lesion related to the Human Papilloma Virus (HPV) infection, and in some cases arising through an HPV independent pathway [3,4].

HPV oncoproteins can induce genetic alterations affecting cell proliferation, the cell cycle, and genomic stability [5,6,7]. In addition, HPV has been proven to induce alterations in the immune microenvironment of infected tissue such as the suppression of immune recognition, promoting acute inflammation and the upregulation of immune checkpoint proteins [8,9,10]. 

The most common treatment for these rare cancers is chemo-radiotherapy and surgery; however, several other strategies are now in clinical trials. As with many other types of cancer, immunotherapy has also been evaluated as a treatment option [10,11,12,13], therefore a comprehensive characterization of tissue biomarkers within the tumor microenvironment of rare cancers is essential.

Tumor-infiltrating lymphocytes (TILs) have shown a crucial role in the anti-tumor immune response as have macrophages, and penile, vulvar, and anal cancer are no exception, with several groups describing the presence of these markers and their role in outcome and survival [9,14,15,16,17,18,19,20,21]. On the other hand, tumor cells can evade immune anti-tumor response using several mechanisms, such as the expression of negative immune checkpoint molecules and the modulation of the tumor microenvironment [22,23,24,25]. The expression of PD-L1 has been demonstrated in penile [26,27], vulvar [28,29] and anal [30,31] carcinomas, with different percentages of positive cells, and these previous reports about PD-L1 expression, including TILs presence, in rare cancer tissues had been evaluated using a classic immunohistochemistry (IHC) [26,27,28,29,30,31]. Even though the IHC is considered as a hallmark for cancer diagnosis, this tool has several limitations, including inter-observer variability, and the fact that it only allows the analysis of one to two biomarkers at a time [32]. Hence, in order to analyze several markers, several histological sections are required, which is challenging when only a small sample of tissue is available [33], or when the cancer type of interest is classified as rare and the tissue sample is limited by the frequency of appearance.

Several tools have been developed for multiplexing targets during histological studies [34]; however, the least expensive is based on multiplexed immunofluorescence (mIF), which advances those challenges by interrogating panels of multiple biomarkers with 5–6 being the most common and up to 9 depending on the instruments and methodology. Due to this advantage, the mIF analysis is a useful tool to characterize the tumor microenvironment, particularly in tumor types with low incidences such as penile, vulvar and anal cancer, and a limited amount of tissue for further studies. 

In this study we describe the technical approach during the implementation of two panels of validated antibodies to evaluate the proliferation rate of tumor cells based on Ki67 signal, tumor infiltrating immune cells by CD8 signal, and by CD68 detection for macrophages, and the tissue localization of the immune checkpoint PD-L1, applying tissue segmentation and cell phenotyping to interrogate the cytokeratin positive and negative compartments in retrospective cases of rare cancer in Chilean patients.

## 2. Materials and Methods

### 2.1. Patients and Case Selection

This is a single-institution retrospective study at Instituto Oncológico Fundación Arturo López Pérez (Santiago, Chile), reviewed and approved by the institutional ethical committee (Prot. 007-RES-ANC-MUL). Deceased patients with histologically proven invasive squamous cell carcinoma of penis, vulva and anus diagnosed in the Institution during 2010–2014 who died because of these diseases between 2014 and 2017 were considered eligible. The respective historical FFPE block was collected from the internal biorepository after the Institutional Ethical Committee approval of the waiver of a signed consent letter. Twenty patient records were retrospectively found, however four cases of penile cancer, one case of vulvar cancer and two of anal cancer had complete clinical records and a suitable tissue sample (Table 1). Clinical and demographic data were retrieved from patient medical records and managed according to privacy regulations.

### 2.2. Sample Processing and Multiplexed Immunofluorescence

For each tumor type, all available tissue cores were requested and examined by a pathologist to confirm the histology and the integrity of the material. Based on hematoxylin-eosin visual inspection, all cores with an area of less than 5% of the tumor were excluded. Immunostaining was carried out as published [35] and each primary cocktail (or panel) was developed using validated antibodies [35,36]. In panel 1: PD-L1 (clone E1L3N), Cytokeratin (clone AE1/AE3), CD68 (clone PG-M1) and CD8a (clone C8/144B). In panel 2: PD-1 (clone D4W2J), Cytokeratin (clone AE1/AE3) and Ki67 (clone MIB-1). Briefly, after antigen retrieval, each whole tissue slide was incubated overnight at 4 °C with the selected panel separately. Isotype-specific HRP-conjugated antibodies and tyramide-based amplification systems (Perkin Elmer, Waltham, MA, USA) were used for signal detection. After co-staining with DAPI, each slide was cover slipped in Prolong Gold mounting medium (Invitrogen, Carlsbad, CA, USA). An index TMA was stained in parallel as quality control. This array included human tissue as naturally positive and negative controls, such as skeletal muscle (negative/noise threshold), tonsil (positive for immune cells), and placenta (positive for PD-L1 immune checkpoint). The dynamic range of positivity for each biomarker was estimated from the ratio between the mean fluorescent signal from a positive control tissue and a negative control tissue. Pearson’s coefficients were calculated between independent staining runs or for single markers to verify concordances between experiments.

### 2.3. Cases and FFPE Cores Considerations

For some analysis considering average values in penile cancer, only cases with more than one FFPE core were considered, however for single case representation all cases were included. For the single case of vulvar cancer, the three FFPE cores available were processed and analyzed in some figures as average values or individual values. For anal cancer, each case and its respective FFPE core was analyzed separately.

### 2.4. Slide Scanning and Image Generation

All slides were scanned at 20× of magnification using an Aperio VERSA 200 microscope (Leica Biosystems, Vista, CA, USA). Exposure times were determined for each target using an index TMA including normal human tissue as positive and negative controls, such as skeletal muscle, tonsil and placenta. The optimal exposure time considered the optimal signal to noise ratio measured for each target and the supervised observation of the operator for the visual positive signal and the expected staining pattern. Later, for each antibody panel, an unsupervised template was designed for the automatic and simultaneous scanning of the fluorescent signal and applied to the tumor whole tissue section. The templates considered the following steps: an initial low-resolution scanning at 1.25× for automatic tissue detection, a second scanning at 5× for fluorescent signal detection (using DAPI); and a final scanning at 20× including sequentially FITC, Cy3, Cy5 and Cy7 filters. Images were saved in scn extension and size varied from 26,976 × 32,249 to 60,440 × 32,249 pixels. After acquisition, all images were revised and exported for further analysis. 

### 2.5. Analysis for Single Objects

Whole-slide scans from the fluorescent-labeled sections were analyzed using QuPath v.0.3.2 software (University of Edinburgh. Edinburgh, Scotland, UK) [37]. DAPI channel was used for cell segmentation using the following settings: Pixel size: 0.5 μm; Background radius: 8.0 μm; Median filter radius: 0.0 μm; Minimum area: 10.0 μm^2^; Maximum area: 200 μm^2^; Threshold: 5.0.

After cell detection, cells were classified using the Object classifier module to determine the number of positive cells for each marker after applying the defined visual cutoff (fluorescent intensity ratio). At least 400 cells from different cores were used to train each object classifier. After every object classifier was trained, they were run for each image using the script editor for batch analysis. Data was exported and analyzed using Microsoft Office tools. The number of total positive cells for each biomarker was determined by the addition of the positive cells for individual biomarkers and the positive ones for more than one biomarker. Percentages were calculated based on the total cell detections.

### 2.6. Analysis for Segmentation and Masking

The pixel classifier from QuPath was trained under manual supervision to detect four different regions: Ignore, corresponding to the sections of the image that had no tissue (glass); Negative, corresponding to regions with auto-fluorescence, mostly red blood cells; Tumor region, defined by Cytokeratin and DAPI positive signal, and Stroma region, corresponding to areas without signal for Cytokeratin, but including DAPI positive objects. For the pixel classifier, 28 fields of view from different tissue images were generated and used for the image training, and the trained classifier was applied in batch or as a single run to each digitized image. The automatic tumor masking was verified visually based on nuclei morphology. Once Pixel classifiers were verified, the tumor and stromal areas were set as annotations and cells within each annotation (tumor or stroma) were classified using the Object Classifier plugin. Data was exported and analyzed in Microsoft Excel. Percentages were calculated based on the total cell detections.

## 3. Results

### 3.1. Immunostaining Verification

**Figure 1:**
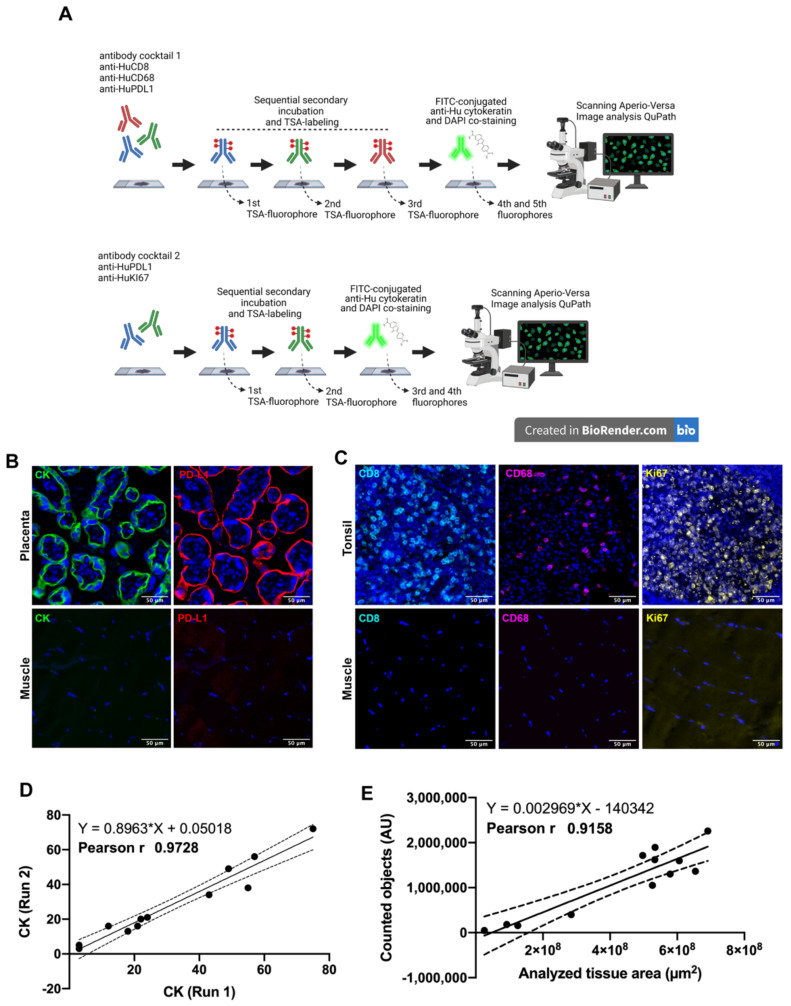
Multiplex Immunofluorescence workflow. In (**A**) after antigen retrieval, each whole tissue slide was incubated with the indicated antibody panel. Sequential signal detection required isotype-specific HRP-conjugated antibodies, tyramide-based fluorophores and digitalization using Aperio VERSA 200 microscope (designed using Biorender). In (**B**,**C**), the staining pattern for positive control tissues are shown. Images are representative for cytokeratin (CK) and PD-L1 in human placenta (**B**). Immune markers CD8 and CD68, and proliferation as Ki67 were verified in human tonsil (**C**). Skeletal muscle was used as negative control. Correlation between two independent staining runs is shown for CK (**D**) as well for the correlation between the scanned area and counted objects (**E**). Scale bar represents 50 µm. Nuclei were stained with DAPI.

Two antibody panels using multiplexed immunofluorescence were designed for the simultaneous detection of CD8+ T-lymphocytes, CD68+ macrophages, Cytokeratin (CK) for tumor cells, PD-L1 immune checkpoint andKi67 as a proliferation marker (Figure 1A). Naturally positive and negative tissues were used as a procedure control: skeletal muscle was used as a negative control while placenta and tonsil tissues were used as positive controls, placenta for antibodies anti-PD-L1 and anti-CK, and tonsil for antibodies anti-Ki67, anti-CD8 and anti-CD68. As we expected, each control tissue showed a very distinctive staining pattern in agreement with previous validation and other reports [35,36]. Placenta showed a membranous and cytoplasmic distribution of PD-L1 and cytokeratin in the trophoblast cells (Figure 1B). Regarding the immune cells, CD8+ lymphocytes and CD68+ macrophages were detected mostly at the medullar and interfollicular area of human tonsils (Figure 1C), and most of the proliferative cells were located within the germinal center as observed through the Ki67 positive signal, which was very well defined and distributed in the nuclear compartment (DAPI co-staining), and only a few cases showed a granular signal at the periphery of the nuclei (Figure 1D). Skeletal muscle did not show fluorescent signal for any tested antibody (Figure 1C,D, lower inserts).

The CK intensity (signal) between two independent staining runs showed a Pearson’s coefficient of 0.97 (Figure 1D), as well as the amount of counted elements using DAPI classifier showing a positive correlation (r 0.91 and *p*-value 0.001) after analyzing several tissue sizes (Figure 1E). Both parameters were representative for other staining procedures.

### 3.2. Multiplexed Immunofluorescence in Rare Cancer Cases Series

Seven retrospective cases with a diagnosis of a rare cancer were selected to interrogate the immune composition and the characteristics of squamous tumor cells in anal, vulvar and penile cancer (Table 1).

**Figure 2:**
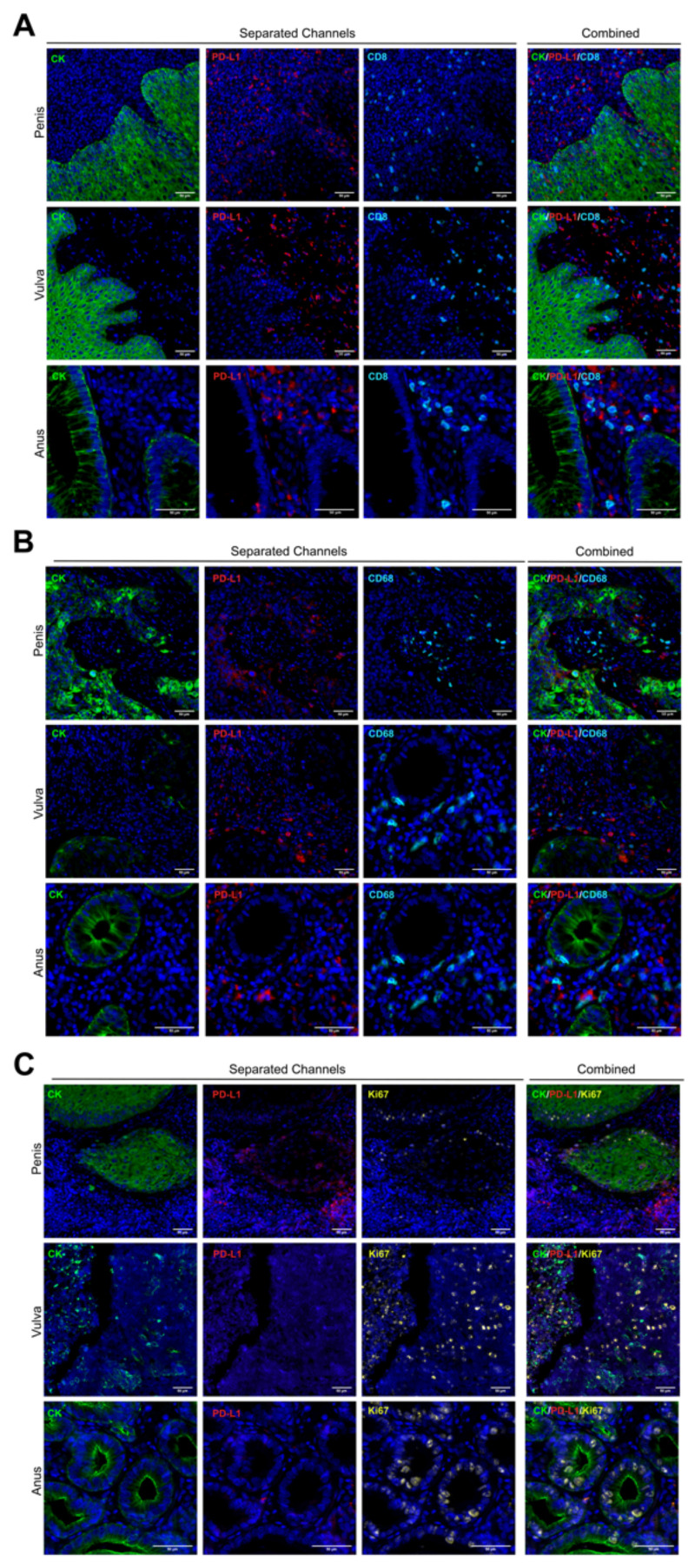
Multiplexed biomarker staining patterns. Whole tissue sections were assessed by multiplexed immunofluorescence. Staining patterns for each biomarker are shown in separated and combined channels, and the images are representative for all cores and tumor types. The cytokeratin CK and PD-L1 area are pseudo-colored in green and red, respectively (**A**–**C**). CD8 (**A**) and CD68 (**B**) are presented in cyan, and Ki67 is was colored in yellow (**C**). 20× of magnification. Scale bar represents 50 µm.

Based on a methodology previously described [35], we stained whole tissue sections of formalin-fixed paraffin-embedded (FFPE) samples from the chosen cases described above. No visual differences in staining patterns were observed for any tumor type (Figure 2). As we expected, tumor cores were reactive for the cytokeratin (CK) antibody clone AE1/AE3, and they showed a membranous-cytoplasmic staining pattern in all cases (Figure 2A–C).

**Figure 3:**
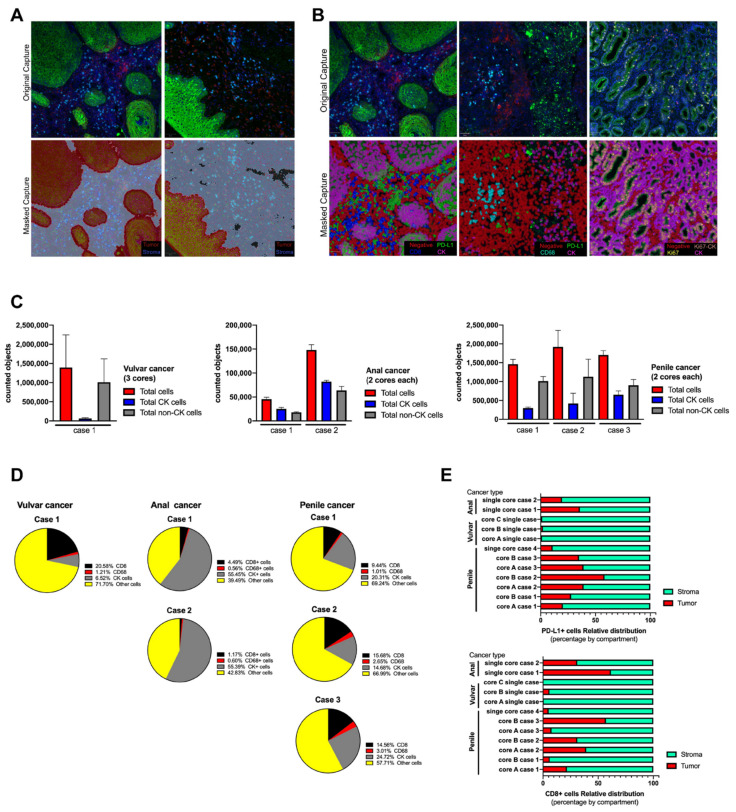
Tissue segmentation and cell phenotyping in cases series of anal, vulvar and penile cancer. Based on QuPath algorithms, the scanned whole tissue sections were masked as tumor and stroma compartments (**A**) using cytokeratin (CK) signal and using single classifiers all cells were labeled and phenotyped (**B**). All counted objects were classified within the tissue section as tumor and non-tumor cells and represented as the average between cores per each case and tumor type (**C**). The relative abundance of infiltrating immune cells and tumor cells was expressed in percentage from the total counted cells (**D**) and calculated for each studied case. After tissue segmentation as tumor (CK positive area) and stroma (CK negative area), CD8 and PD-L1 signal was counted as individual and its distribution within both compartments was expressed as a percentage (**E**). To test variability, the anal cancer cases were processed twice, and for penile cancer only cases with more than one core were included.

PD-L1 protein was mostly positive in penile tumor cells but with very low detection in anal and vulvar squamous tumor cells (Figure 2). Several non-tumor cells (CK negative) within the stromal area showed a strong signal for PD-L1, but without a clear trend for any tumor types or analyzed core (Figure 2 and Figure 3E). Lymphocytes and macrophages were observed surrounding and infiltrating the tumor compartment for the three tumor types; however, TILs showed a preferential stromal distribution in vulvar cancer (Supplementary Figure S1). Based on the Ki67 signal, a broad range of proliferative cells were observed, without a significant mean trend for any cancer type (Figure 2C).

### 3.3. Image Analysis by Tissue Segmentation and Cell Phenotyping

As a first approach, two antibody panels for mIF were designed and considered PD-L1 and cytokeratin as common targets to be used as reference to identify common elements and perform association between biomarkers detected in different slides. 

For the cell segmentation analysis, all images were analyzed using the positive cell detection module of QuPath software and DAPI as reference for single counts [37]. No significant differences were observed when an area of 100µm2 was manually counted and compared with the counts using QuPath, and the estimated error for the automated single cell segmentation was 2–5% (data not shown). After cell segmentation by nuclei detection, QuPath object classifier module generated a single-color mask for each targeted biomarker. Positive cells were masked in color based on QuPath color code (Figure 3A,B). Tumor and stroma compartments were built using the positive and negative areas for CK signal, respectively, and masked as shown (Figure 3A). The accuracy increased with the intensity of the fluorescent signal and the depth of the selected pixels. A similar observation was verified with the cell classifier (Figure 3B), however, because the area per single cell was smaller, the accuracy depended on the initial DAPI detection. Each tissue section was analyzed twice, “one by one” and “in batch” mode, and the masking images were comparable with each other. For each biomarker, the estimated error of counted cells between cores was below 10%, and the highest variability was observed for the proportion of CK+ cells with respect to the total cells counted within the stained section per core (data not shown).

After defining the settings for an automated tissue segmentation and cell phenotyping, all images were processed and the abundance of each cell type was counted per scanned area and expressed as the average between cores for the same case (Figure 3C,D). All sections showed variability in the number of counted objects without affecting the trend for each tumor case (Supplementary Figure). Regarding immune cells, CD68 macrophages were the less abundant object for any analyzed tissue (Figure 3D) which was related to its biology per se and not to the intensity of signal as in cytokeratin. On the other hand, CD8 TILs were frequently detected and their distribution was preferentially within the stroma compartment (Figure 3E and Supplementary Materials). Very few cells showed reactivity for PD-L1, which limited the analysis, however this immune checkpoint did not show a predominant distribution or compartmentalization between stroma and tumor. Penile and anal cancer cases showed a clear expression of PD-L1 and the variability between tissue cores was smaller than the observed distribution of CD8 cells (Figure 3D). For a better understanding, the cell phenotyping analysis was applied in tumor cells using the combination of key biomarkers, such as PD-L1 and Ki67, and expressed as a percentage of relative abundance within a selected region (Table 2); the counted objects allowed to express it as an objective score and was able to count less than 1% of a specific cell phenotype within the total scanned area.

## 4. Discussion

The Society for Immunotherapy of Cancer (SITC) has provided the fundamentals of best practices for multiplex immunohistochemistry (mIHC) and immunofluorescence (mIF) staining and validation, and suggested that mIHC/mIF technologies are becoming standard tools for biomarker studies and are likely to enter routine clinical practice in the near future [38]. For that reason, a careful assay optimization and validation is required to ensure that outputs are robust and comparable across laboratories as well as potentially across mIHC/mIF platforms.

This study represents the beginning of the use of multiplexing and its validation process developed by a Chilean cancer center based on the recommendations of the SITC, and included an objective and reliable approach for the characterization of a tumor microenvironment in FPEE samples from invasive squamous cell carcinomas of the penis, vulva and anus. These types of tumors are classified as rare for their low incidence in the population compared to other types of malignancies [1,39,40]. Since the number of samples is always limited for this type of cancer, we have developed a multiplex immunofluorescence analysis that allowed us to obtain more information from a single histological section.

After reviewing three different case series of two anal cancers (single tissue core each), of one vulvar cancer (three tissue cores), and of four penile cancers (two cores and one single core), using a single biomarker for cell phenotyping may reduce the possibility for the researcher and the pathologist to detect the natural variability between cores and cases, and the combination of biomarkers may highlight the differences between patients and even between tissue cores from a same patient.

As far as we know, this is one of the first studies to analyze FFPE samples from rare cancers in Chilean patients by multiplexed immunofluorescence (mIF). Previously, studies based on image analysis from FFPE samples from these types of cancer were performed using classic IHC, which has a limitation regarding the number of biomarkers to analyze and a high inter-observer variability [32,33].

The mIF facilitates the study of these rare cancers because it enables us to analyze the co-expression of different biomarkers for a better understanding of the tumor microenvironment, identifying different cell phenotypes combining biomarkers and maintaining the spatial distribution for each detected element [41]. Using an open-source software such as QuPath allows anyone, including research laboratories with limited resources, to collect a large amount of data, from single counts, location and distribution and regional abundance using positive pixel training to identify the target of interest [42,43,44,45]. Based on the software’s plugins, we analyzed the number of positive cells for each biomarker in the whole tissue section and ran the analysis in batch, which reduced time and variability inter-reader [45,46]. After managing the single cell identifier, larger areas of tissue can be analyzed by specific signal masking, such as tumor nest and stroma. The QuPath plugin, through a supervised training, identifies pixels with a pan-cytokeratin antibody signal, segregating them into pixels with and without information to define an algorithm for the automatic tissue masking and the execution of a secondary analysis, such as counting single elements within the selected area as a regular machine learning operation [47,48].

The selection of biomarkers used in this work was based on their clinical relevance and role in tumor microenvironment adaptation. Tumor-infiltrating CD8 lymphocytes are considered as the major inflammatory component in almost all known solid tumors and are responsible for killing tumor cells by their cytotoxic activity [49,50] In addition, these TILs may become exhausted because they are exposed to a sustained immune suppressive environment, such as tumor cells and myeloid cells expressing PD-L1. As reported by our collaborators and other researchers, using immune checkpoint blockers may reinvigorate some cytotoxic CD8 T cells and restore their effector role against several tumor types [51,52]. In addition, radiotherapy is frequently used to treat these type of cancers, and recently an abscopal effect after irradiation regimens was described in preclinical models where CD8 T cells were shown to be crucial in improving the response to immunomodulatory monoclonal antibodies, such as anti-PD1 or anti-CD137 [52].

Different mechanisms of immune evasion have been studied in penile [11,21,53,54] vulvar [14,18,20,55] and anal cancers [17,19], and included immune checkpoint proteins and exhausted phenotypes for infiltrating-lymphocytes, suggesting that patients with these type of tumors can benefit from anti PD-L1 therapy [9,27,56,57,58,59]. Currently, there are several clinical trials ongoing for the use of immune therapy targeting PD-L1 (or its receptor PD-1) as treatment for penile, vulvar or anal cancer [60,61,62,63,64,65,66,67,68,69]. Therefore, multiplexed immunofluorescence and an objective analysis of the tumor microenvironment may contribute significantly to improve the understanding of different therapeutic targets when the access to biological material is reduced or limited.

Flow cytometry has been the most extended tool to characterize specific cell phenotypes based on the multiple detection of surface and intracellular biomarkers, in particular for non-tumor cells within the tumor microenvironment, such as infiltrating lymphocytes [70] and tumor-associated macrophages (TAM) [71]. We did not include specific phenotypes, such as M1 or M2 macrophages, because of the lack of validated antibodies for histochemistry or the complexity of combining some of them, and CD68 alone may be considered as an indicator of TAM-like cells. Other limitations of this study include the small number of analyzed cases which may affect the ability of the trained algorithms to recognize a variable related to a specific-tissue type, in particular in the only one case of vulvar cancer. In addition, we were aware of the absence of statistical power, which limited the analysis but it does not invalidate the histological findings and developed tools. Finally, we determined the localization of single objects but we did not carry out algorithms to measure distance between objects, which has also been described as being determinant in tumor microenvironment characterization [72].

## 5. Conclusions

We have described two multiplex antibody panels to identified biomarkers and developed a supervised machine learning analysis based on positive pixels that is able to do tissue segmentation, cell phenotyping and object counting for the objective and automated scoring of immune checkpoint proteins, tissue immune infiltration and proliferation in FFPE samples from solid tumors such as penile, vulvar, or anal cancer.

## Figures and Tables

**Table 1:** Clinical and demographic information of the case series of anal, vulvar and penile cancer included in this research.

Tumor Type	Patient ID *	FFPE Block	Age at Diagnosis	Gender	Vital Status	Histology	Grade	Stage	TNM	Other Conditions
Penile	Case 1	Core A	72	Male	Deceased	Squamous cell carcinoma	Well differentiated	III	T3N2M0	None
		Core B								
	Case 2	Core A	57	Male	Deceased	Squamous cell carcinoma	Poorly differentiated	III	T3N1M0	None
		Core B								
	Case 3	Core A	61	Male	Deceased	Squamous cell carcinoma	Moderately differentiated	IV	T4N2M1	None
		Core B								
	Case 4	Core A	31	Male	Deceased	Squamous cell carcinoma	Moderately differentiated	IV	T4N1M1	HIV infection
Vulva	Case 1	Core A	67	Female	Deceased	Squamous cell carcinoma	Poorly differentiated	IV	T4N2M1	Hypertension
		Core B								
		Core C								
Anal	Case 1	Core A	69	Female	Deceased	Squamous cell carcinoma	Poorly differentiated	II	T2N1M0	Hypertension
	Case 2	Core A	74	Female	Deceased	Squamous cell carcinoma	Moderately differentiated	III	T3N1M0	Meningioma

* All cases were previously anonymized and segregated by affected organ, number and respective tissue cores (FFPE block).

**Table 2:** Tumor cells phenotyping and multiple biomarker labeling.

Tumor Cell Phenotyping *			Counted Cells	Counted CK (+)	% Ki67 (−)/PD-L1 (−)	% Ki67 (+) Cells	% PD-L1 (+) Cells	% Ki67 (+)/PDL1 (+)
penile cancer	Case 1	Core A	1,301,254	306,091	95.15	4.13	1.39	0.60
		Core B	1,365,949	294,761	95.80	4.13	0.12	0.04
	Case 2	Core A	1,894,818	400,175	69.79	28.19	3.63	0.51
		Core B	1,622,523	897,117	93.09	6.67	0.37	0.10
	Case 3	Core A	1,597,643	679,128	96.28	3.49	0.28	0.03
		Core B	1,713,451	839,073	96.90	2.92	0.24	0.05
	Case 4	Single core	181,154	32,011	93.89	4.62	0.84	2.48
vulvar cancer	Case 1	Core A	2,258,207	60,982	96.22	3.35	0.02	0.48
		Core B	402,220	46,374	99.80	0.06	0.01	0.15
		Core C	1,052,697	27,993	99.61	0.06	0.01	0.34
anal cancer	Case 1	Single core	49,977	37,524	72.04	23.81	1.62	8.00
	Case 2	Single core	158,127	89,776	93.44	5.34	0.60	1.94

* Phenotyping of tumor cells was performed per core and tumor type. Cytokeratin (CK) positive cells were detected, classified and masked for the single or double detection of Ki67 and PD-L1 signal. All possible phenotypes were tagged and counted. Relative abundance was expressed as percentage. (+) Positive, (−) Negative. Percentage was calculated from the total number of CK+ cells.

## Data Availability

The data presented in this study are available on request from the corresponding author. The data are not publicly available due to ethical restrictions.

## Supplementary Materials

The following supporting information can be downloaded at: https://www.mdpi.com/article/10.3390/cancers14112815/s1, Figure S1: Counted elements per case and tissue core.
