# Supplementary material for: Simultaneous and Spatially-Resolved Analysis of T-Lymphocytes, Macrophages and PD-L1 Immune Checkpoint in Rare Cancers
Source: Cancers (Basel). 2022 Jun 6;14(11):2815. doi: 10.3390/cancers14112815 (PMC9179863; doi:10.3390/cancers14112815)
Supplement: Supplementary file 1 [file cancers-14-02815-s001.zip › cancers-1738973-supplementary.pdf]

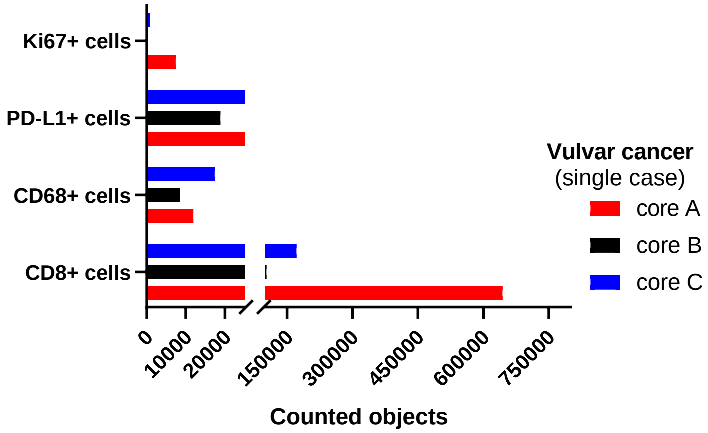

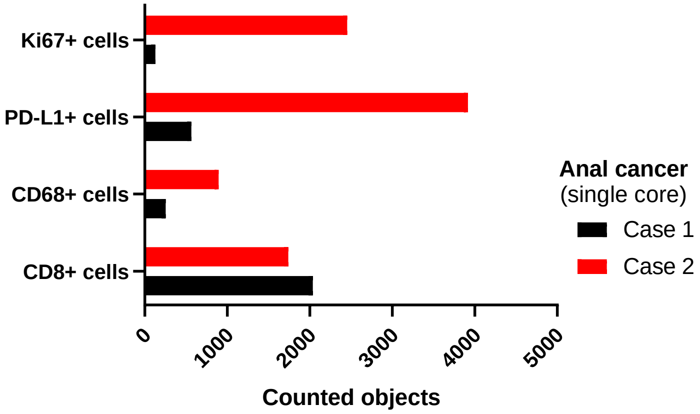

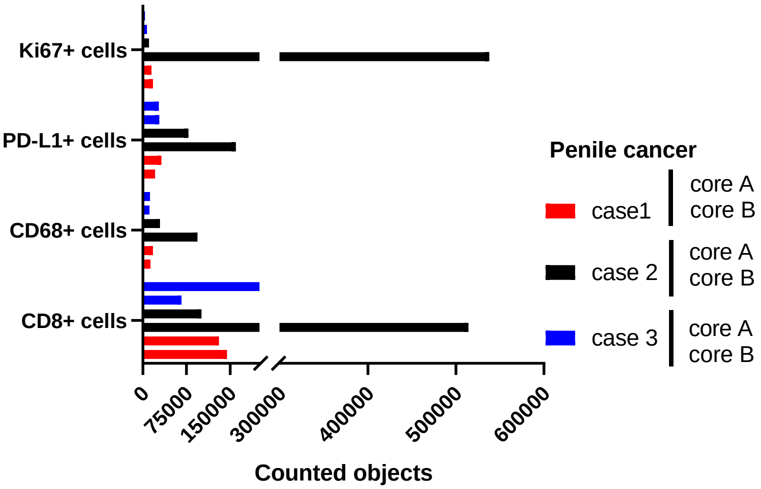

Supplementary figure S1: Counted elements per case and tissue core. Single cells were masked using DAPI and classified using one classifier at time (positive signal). Counted objects represent the total number for each phenotype as a unique label, not considering double or triple positive cells.
